# Outcomes of an intermediate respiratory care unit in the COVID-19 pandemic

**DOI:** 10.1371/journal.pone.0243968

**Published:** 2020-12-16

**Authors:** Javier Carrillo Hernandez-Rubio, Maria Sanchez-Carpintero Abad, Andrea Yordi Leon, Guillermo Doblare Higuera, Leticia Garcia Rodriguez, Carmen Garcia Torrejon, Alejandro Mayor Cacho, Angel Jimenez Rodriguez, Mercedes Garcia-Salmones Martin

**Affiliations:** 1 Department of Pulmonology and Respiratory Medicine, Rey Juan Carlos University Hospital, Madrid, Spain; 2 Department of Pulmonology and Respiratory Medicine, Infanta Elena University Hospital, Madrid, Spain; 3 Department of Intensive Care Medicine, Infanta Elena University Hospital, Madrid, Spain; 4 Department of Anesthesiology, Infanta Elena University Hospital, Madrid, Spain; 5 Department of Internal Medicine, Infanta Elena University Hospital, Madrid, Spain; Ohio State University, UNITED STATES

## Abstract

**Background:**

15% of COVID-19 patients develop severe pneumonia. Non-invasive mechanical ventilation and high-flow nasal cannula can reduce the rate of endotracheal intubation in adult respiratory distress syndrome, although failure rate is high.

**Objective:**

To describe the rate of endotracheal intubation, the effectiveness of treatment, complications and mortality in patients with severe respiratory failure due to COVID-19.

**Methods:**

Prospective cohort study in a first-level hospital in Madrid. Patients with a positive polymerase chain reaction for SARS-CoV-2 and admitted to the Intermediate Respiratory Care Unit with tachypnea, use of accessory musculature or Sp_O2_ <92% despite Fi_O2_> 0.5 were included. Intubation rate, medical complications, and 28-day mortality were recorded. Statistical analysis through association studies, logistic and Cox regression models and survival analysis was performed.

**Results:**

Seventy patients were included. 37.1% required endotracheal intubation, 58.6% suffered medical complications and 24.3% died. Prone positioning was independently associated with lower need for endotracheal intubation (OR 0.05; 95% CI 0.005 to 0.54, p = 0.001). The adjusted HR for death at 28 days in the group of patients requiring endotracheal intubation was 5.4 (95% CI 1.51 to 19.5; p = 0.009).

**Conclusions:**

The rate of endotracheal intubation in patients with severe respiratory failure from COVID-19 was 37.1%. Complications and mortality were lower in patients in whom endotracheal intubation could be avoided. Prone positioning could reduce the need for endotracheal intubation.

## Introduction

On March 11, 2020, the World Health Organization declared the novel COVID-19 outbreak a global pandemic [1]. More than three million cases had been reported worldwide by the end of the first week in May, of which 221,000 had been declared in Spain [2]. The spectrum of this disease caused by the SARS-CoV-2 coronavirus ranges from a common cold to a severe pneumonia defined according to American Thoracic Society criteria [3] in a not negligible 15% of patients [4]. In our setting, the rapid increase in the incidence of COVID-19 and consequent saturation of the capacity of the intensive care units (ICUs) led to a significant role for intermediate respiratory care units (IRCUs) in the management of these patients, with the principal purpose of reducing the need for endotracheal intubation (ETI) using non-invasive respiratory support.

The efficacy of high-flow nasal cannula (HFNC) and non-invasive mechanical ventilation (NIV) in adult respiratory distress syndrome (ARDS) have been previously investigated as therapies that could reduce intubation rate and mortality [5–7]. However, the use of these respiratory support therapies beyond the stablished time or severity window of the ARDS could lead to an increase in mortality [8], with failure rates in moderate or severe forms between 38% and 80% respectively [9, 10]. Hence the importance of adequate selection of patients and early access to ETI in the absence of response.

The objective of the study is to describe the ETI rate in patients with severe respiratory failure due to COVID-19 managed in an IRCU, the efficacy of the respiratory support and pharmacological treatments, and the medical complication and mortality rates.

## Material and methods

**Fig 1:**
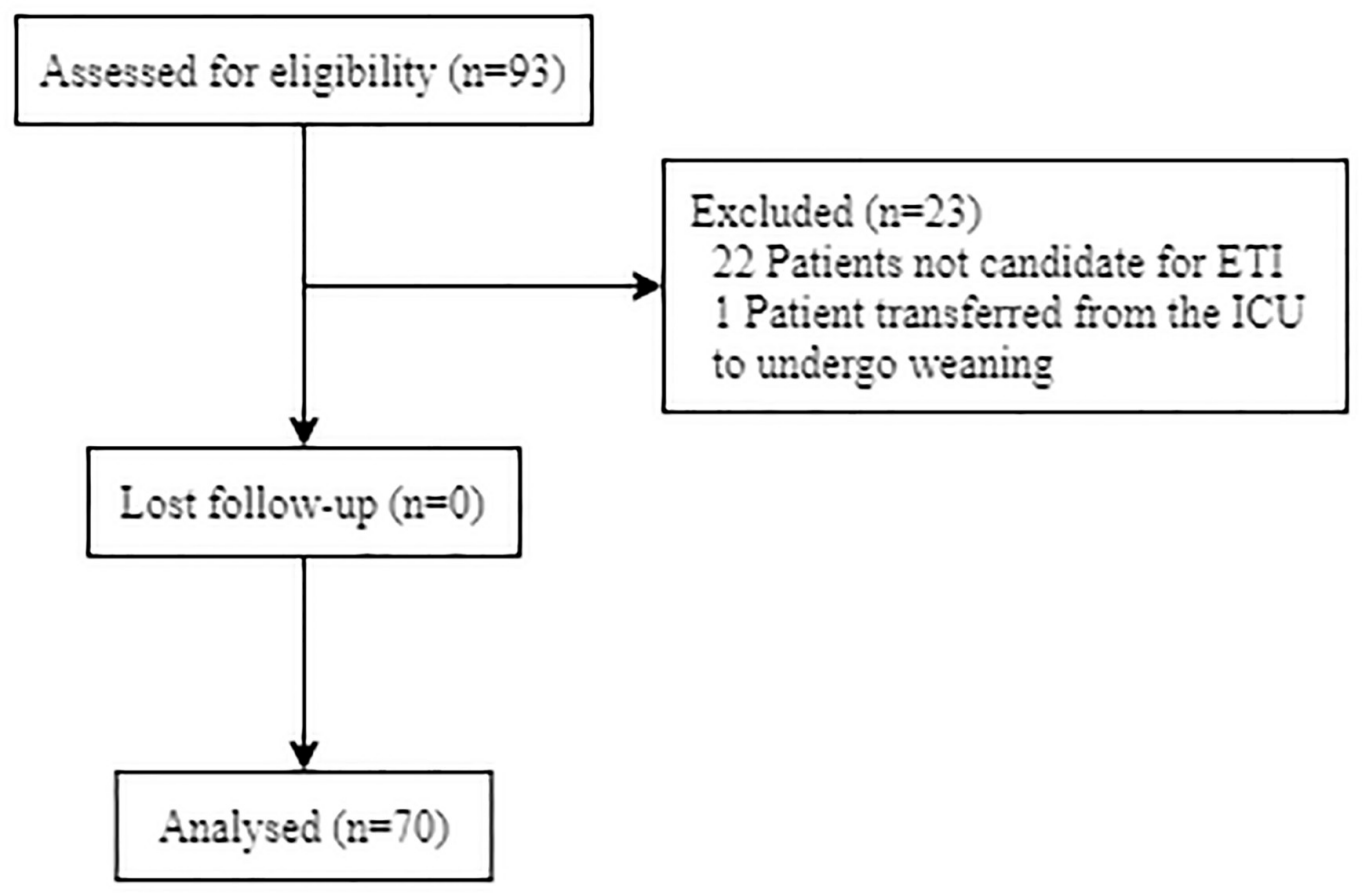
Flow diagram of patient selection. ETI: Endotracheal intubation, ICU: intensive care unit.

This is a prospective cohort study conducted in an 11-bed IRCU led by a team of pneumologists with support from intensive care and anesthesia specialists in the first-level Infanta Elena University Hospital, Madrid, Spain. Inclusion criteria were the following: adult patients with a positive PCR for COVID-19 and admission to the IRCU with at least one of the following: respiratory rate (RR) > 30 breaths·minute^-1^, severe dyspnea, use of accessory muscles or Sp_O2_ <92% despite Fi_O2_ >0.5 oxygen therapy. Patients were included between March 6 and April 8, 2020 and were follow-up for a 28-day period. Patients not candidate for ETI according to the ethics committee document created and approved in March 2020 and patients transferred from the ICU to undergo weaning were excluded from the study (Fig 1).

Specific COVID-19 pharmacological treatment choice was stablished at the discretion of the prescribing specialist following the center’s protocol (S1 Table).

In patients presenting a Sp_O2_ <92% despite Fi_O2_> 0.5 without a RR of > 30 breaths·minute^-1^ or use of accessory muscles upon admission to the IRCU, treatment with HFNC (AIRVO 2, Fisher and Paykel healthcare) was started with an initial flow of 60 liters/minute, a temperature of 37.0°C and a Fi_O2_ between 0.5 and 1 with the objective of a Sp_O2_> 92%.

In patients with RR> 30 breaths·minute^-1^, severe dyspnea or use of accessory muscles, support was started with IRCU or home ventilators (V60 Philips Respironics, Vivo 55 Breas, Vivo 60 Breas and Astral 150 Resmed) in CPAP or bilevel pressure mode, titrating the positive end expiratory pressure (PEEP) to achieve an Sp_O2_> 92% with the lowest possible Fi_O2_ and the support pressure (SP) to reduce the RR <30 breaths·minute^-1^, the use of accessory muscles and the degree of dyspnea. In all cases, it was considered a priority to achieve a tidal volume of less than 6–8 ml·min^-1^·kg^-1^ of ideal body weight [11]. The use of Helmet was preferred for its advantages in terms of efficacy [5] and safety [12] in accordance with the recommendations of our scientific society [13]. At the discretion of the prescribing clinician, the patient was pronated in the first 12 hours of admission to the IRCU between 1 and 3 times per day for 60 minutes or as long as the patient could tolerate.

**Fig 2:**
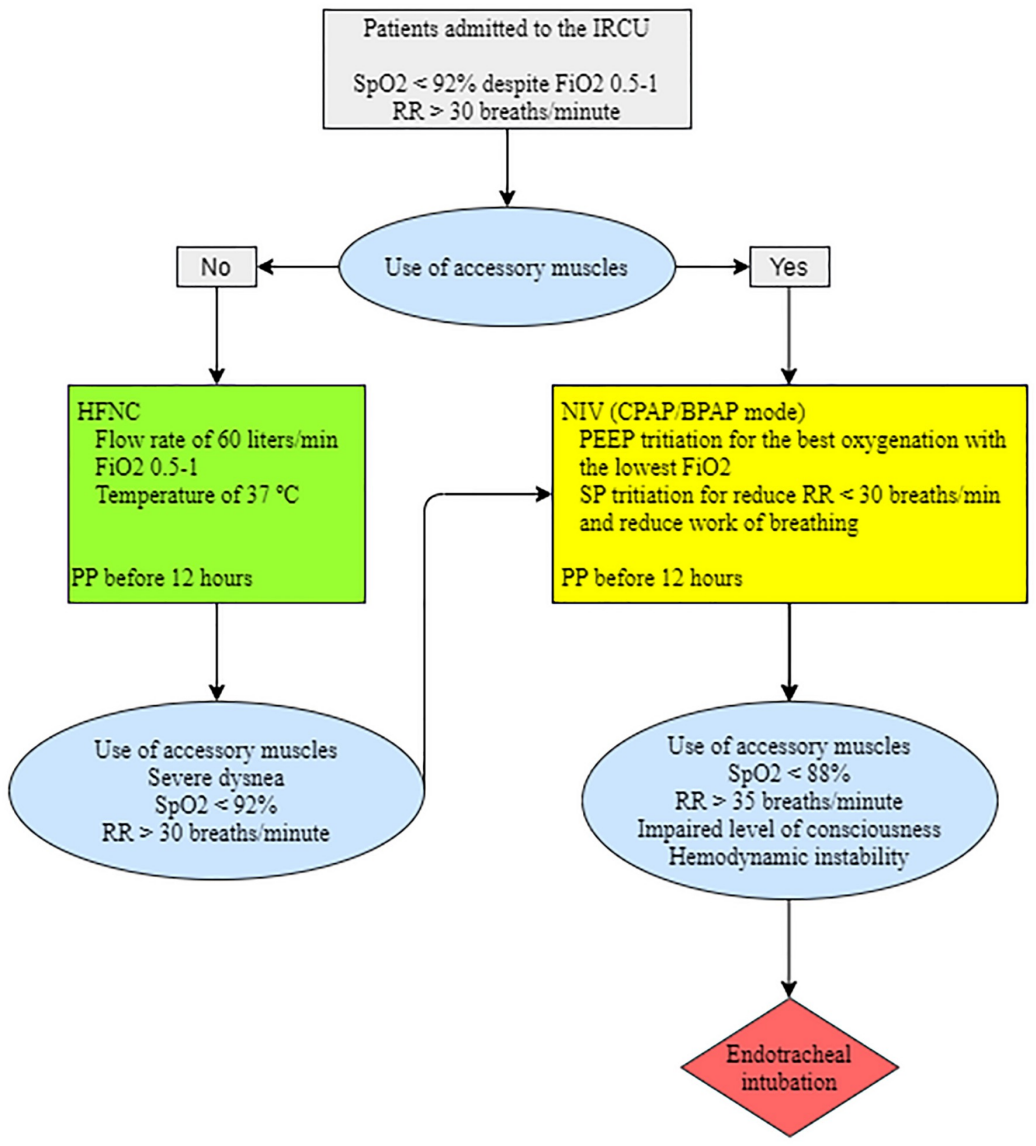
Diagram of respiratory support. IRCU: Intermediate Respiratory Care Unit, RR: respiratory rate, HFNC: high flow nasal cannula, PP: prone positioning, NIV: non-invasive ventilation, CPAP: continuous positive airway pressure, BPAP: bilevel positive airway pressure, PEEP: Positive end-expiratory pressure, SP: support pressure.

Discharge to conventional hospitalization occurred when the patient presented an Sp_O2_> 92% with Fi_O2_ <0.5, RR <30/min, and had no evidence of dyspnea or use of accessory muscles. ETI was performed if Sp_O2_ <88%, RR> 35 breaths·minute^-1^, impaired level of consciousness or hemodynamic instability, despite 1–2 hours of respiratory support with a Fi_O2_> 0.5. The respiratory support algorithm is illustrated in Fig 2.

Upon admission to the IRCU, a chest radiograph was performed and a blood sample was taken for the analysis of pH, partial pressure of O2 (Pa_O2_) and CO2 (Pa_CO2_), and a complete biochemistry including creatinine, urea, bilirubin, liver enzymes, profile ionic, ferritin, procalcitonin, interleukin 6 (IL-6), blood count and coagulation. Age, sex, body mass index (BMI), the Charlson index and the Simplified Acute Physiology Score II (SAPS II), the choice of treatment, the highest degree of respiratory support required, the parameters of PEEP, SP and whether the patient was set in the prone position (PP) were recorded. The incidence and number of grade III, IV, and V medical complications were recorded according to the Common Terminology Criteria for Adverse Events (CTCAE) v5.0, as well as the intubation rate, and 28-day mortality.

The study was approved by the Research Ethics Committee of the Jiménez Díaz Foundation (code EO 081–20_HIE). The need for consent was waived by the ethics committee.

Assuming an intubation rate of 60% (9,10) in patients with moderate or severe ARDS treated with HFNC or NIV, we calculated that a sample size of at least 59 patients would be required with a two tail alpha risk of 0.05 and a statistical power of 80% in order to detect a difference in the ETI rate of 20%, which we consider clinically relevant. A follow-up loss rate of 20% was estimated.

Descriptive statistics were obtained for all study variables. The Shapiro-Wilk test was used to verify the normality. Normally distributed quantitative variables are expressed as mean (standard deviation) or as median (interquartile range). Categorical variables are expressed as absolute and percentage value. The characteristics of the groups of intubated and non-intubated patients and deceased and non-deceased patients were compared using the Student’s t-test and the Mann-Whitney U test for quantitative variables and the Chi-square test and Fisher’s exact test for categorical variables. Multivariate logistic regression was performed to determine whether there were any independent predictors of ETI. Kaplan-Meier survival curves were constructed, and a log-rank analysis was conducted. We determined predictors of 28-day mortality rate using Cox proportional hazard models. For all statistical analyzes, a p<0.05 was considered significant. Data were analyzed with IBM SPSS Statistics for Windows, Version 22.0. Armonk, NY: IBM Corp.

## Results

From March 6 to April 8, 2020, 93 patients were screened, of whom 70 patients were finally included in the analysis.

The median age was 60 years (range: 50.7–71.2) and 77.1% were male patients. 55.7% of patients ascertained were obese. On admission, the median Pa_O2_/Fi_O2_ was 83 mmHg (range: 55 to 142) and the mean SAPS II 34.3 ± 7.9 (SD). 55.7% of the patients required HNFC and 27.1% required NIV in CPAP or BPAP mode, and frequent Helmet use (63.2%). 37.1% of patients required ETI, 58.6% of patients suffered major medical complications, and mortality was 24.3%. The baseline characteristics of the group that required ETI and the group that did not require it are summarized in Table 1.

**Table 1:** Clinical and biochemical characteristics of the patients who required endotracheal intubation and those who did not.

	Intubated (n = 26)	Not intubated (n = 44)	p-value
Age, years*	62.5 (49.7 to 73.0)	58.5 (51.0 to 71.0)	0.66
Male sex, No. (%)	20 (76.9)	31 (77.3)	0.97
Body mass index, kg·m^-2.^*	31.2 (29.8 to 34.9)	31.3 (29.4 to 34.3)	1
Charlson Index score*	3.0 (1.0 to 5.0)	2.0 (1.0 to 4.0)	0.91
Pa_O2_/Fi_O2_, mmHg*	83.5 (51.5 to 141.7)	80.0 (64.0 to 143.0)	0.87
pH	7.45 (0.06)	7.40 (0.05)	0.01
Pa_CO2_, mmHg*	35.8 (6.4)	39.9 (6.8)	0.03
SAPS II score	33.7 (8.2)	34.7 (7.8)	0.62
Lymphocytes cells·L^-1^	807 (467)	940 (249)	0.22
D dimer, μg·ml^-1,^*	309 (256 to 736)	472 (261 to 936)	0.83
Ferritin, ng·ml^-1,^*	1752 (951 to 2939)	1327 (708 to 1988)	0.75
Procalcitonin, ng·ml^-1,^*	0.23 (0.16 to 0.47)	0.13 (0.06 to 0.24)	0.03
Interleukin 6, pg·ml^-1,^*	150 (54 to 1354)	54.2 (23.4 to 88.7)	0.02

SAPS II, Simple Acute Physiologic Score II.

*Data expressed as median (interquartile range).

The levels of pH, procalcitonin, and IL-6 were significantly higher in the group that required endotracheal intubation (p <0.05), while the Pa_CO2_ was higher in the group of patients that did not require intubation (p <0.05). No other significant differences for the analyzed variables were observed between both groups (Table 1).

Patients who required endotracheal intubation underwent treatment with oxygen therapy and PP in lesser proportion (p <0.05), with more frequent use of acetylcysteine, azithromycin and betaferon and less use of cyclosporine (p <0.05). No significant differences were observed in the type of respiratory support received, the ventilation parameters, the type of interface or in the rest of the pharmacological treatment used (Table 2).

**Table 2:** Characteristics of the support and pharmacological treatment of patients who required endotracheal intubation and those who did not.

	Intubated (n = 26)	Not intubated (n = 44)	p-value
Oxygen therapy, No. (%)	1 (3.8)	11 (25.0)	0.02
High Flow nassal cannula, No. (%)	18 (69.2)	21 (47.7)	0.08
CPAP, No. (%)	2 (7.7)	5 (11.4)	1
BPAP, No. (%)	5 (19.2)	7 (15.9)	0.75
Helmet, No. (%)^§^	4 (57.1)	8 (66.6)	1
CPAP level, cmH_2_O*	14.0 (13.0 to 14.0)	15.0 (13.5 to 15.0)	1
IPAP level, cmH_2_O	17.2 (2.5)	18.8 (2.6)	0.30
EPAP level, cmH_2_O*	10.0 (10.0 to 13.0)	11.0 (10.0 to 12.0)	1
Prone positioning, No. (%)	6 (23.1)	26 (59.1)	0.003
Treatment received			
Acetylcysteine, No (%)	21 (80.8)	23 (52.3)	0.01
Azithromycine, No. (%)	13 (50.0)	9 (20.5)	0.01
Betaferón, No. (%)	15 (57.7)	7 (15.9)	<0.001
Cyclosporine, No. (%)	5 (19.2)	25 (56.8)	
Hydroxychloroquine, No. (%)	24 (92.3)	44 (100)	0.002
Lopinavir/ritonavir, No. (%)	26 (100)	42 (95.5)	0.13
Methylprednisolone (bolus 250 mg),	11 (42.3)	22 (50.0)	0.27
No. (%)			0.53
Methylprednisolone (≥ 1 mg·kg^-1^·day^-1^), No. (%)	16 (61.5)	35 (79.5)	0.10
Tocilizumab, No. (%)	18 (69.2)	31 (70.5)	0.91

CPAP, continuous positive airway pressure; IPAP, inspiratory positive airway pressure; EPAP, Expiratory Positive Airway Pressure; BPAP, bilevel positive airway pressure.

*Data expressed as median (interquartile range).

^§^From the total of patients who received non-invasive ventilation (CPAP or BPAP mode).

Patients who required endotracheal intubation suffered a higher incidence and number of complications (p <0.05), with a higher proportion of myocardial injury, hypertension, acute kidney failure, bacteremia, septic shock, nosocomial pneumonia, bronchial obstruction, anemia, thrombopenia, and cutaneous ulcers. The mortality in patients who required intubation was significantly higher than those who did not require intubation (p <0.001) (Table 3).

**Table 3:** Complications and mortality of patients who required intubation and those who did not.

	Intubated (n = 26)	Not intubated (n = 44)	p-value
Myocardic injury, No. (%)	8 (30.8)	4 (9.1)	0.046
Highest cTnT value, media (DE), μg·L^-1^	0.151 (0.06)	0.052 (0.02)	0.03
QT prolongation, No. (%)	3 (11.5)	7 (15.9)	0.73
*De novo* supraventricular tachycardia, No. (%)	3 (11.5)	1 (2.3)	0.15
Acute pulmonary embolism, No. (%)	1 (3.8)	3 (6.8)	1
Hypertension, No. (%)	7 (26.9)	3 (6.8)	0.03
Hypotension, No. (%)	2 (7.7)	2 (4.5)	0.63
Acute kidney failiure, No. (%)	9 (34.6)	2 (4.5)	0.002
Bacteraemia, No. (%)	4 (15.4)	0 (0)	0.01
Septic shock, No. (%)	5 (19.2)	0 (0)	0.006
Hospital acquired pneumonia, No. (%)	5 (19.2)	0 (0)	0.006
Intravascular catheter infection, No. (%)	3 (11.5)	0 (0)	0.052
Fungal infection, No. (%)	2 (7.7)	0 (0)	0.14
Cytomegalovirus infection, No. (%)	1 (3.8)	0 (0)	0.38
Agitation, No. (%)	3 (11.5)	1 (2.3)	0.15
Seizure, No. (%)	0 (0)	1 (2.3)	1
Bronchial obstruction, No. (%)	9 (34.6)	0 (0)	<0.001
Pneumothorax, No. (%)	3 (11.5)	0 (0)	0.052
Abnormal liver function, No. (%)	6 (23.1)	7 (15.9)	0.54
Hyoperkalemia, No. (%)	1 (3.8)	1 (2.3)	1
Hypokalemia, No. (%)	1 (3.8)	1 (2.3)	1
Hypernatremia, No. (%)	1 (3.8)	0 (0)	0.38
Hyponatremia, No. (%)	0 (0)	1 (2.3)	1
Hypocalcemia, No. (%)	1 (3.8)	1 (2.3)	1
Anemia, No. (%)	9 (34.6)	2 (4.5)	0.002
Thrombocytopenia, No. (%)	4 (14.5)	0 (0)	0.01
Gastrointestinal bleeding, No. (%)	1 (3.8)	1 (2.3)	1
Skin ulceration, No. (%)	4 (15.4)	0 (0)	0.01
Major complications, No. (%)	21 (80.8)	20 (45.5)	0.001
Number of major complications*	4.5 (1.0 to 6.7)	0.0 (0.0 to 1.7)	0,002
Mortality, No. (%)	14 (53.8)	3 (6.8)	<0,001

cTnT, cardiac troponin T.

*Data expressed as median (interquartile range).

A logistic regression model was designed to search for predictive variables of ETI following the backwards method. The variables age, sex, Pa_O2_/Fi_O2_, lymphocytes, D-dimer, procalcitonin, IL-6, pronation, the use of acetylcysteine, azithromycin, betaferon and cyclosporin were included in the model. PP was observed to be independently associated with the need for intubation (adjusted OR of 0.05, 95% CI 0.005 to 0.54, p = 0.001) (the baseline characteristics of patients who received PP and those who did not are summarized in the S3 Table) as well as IL-6 values greater than 1000 pg/ml (adjusted OR of 65.2, IC95% 3.5 to 1198, p = 0.005). In the group of patients with high levels of IL-6 (> 1000 pg·ml^-1^), no significant difference in intubation rate or mortality was observed between those who received tocilizumab and those who did not.

**Fig 3:**
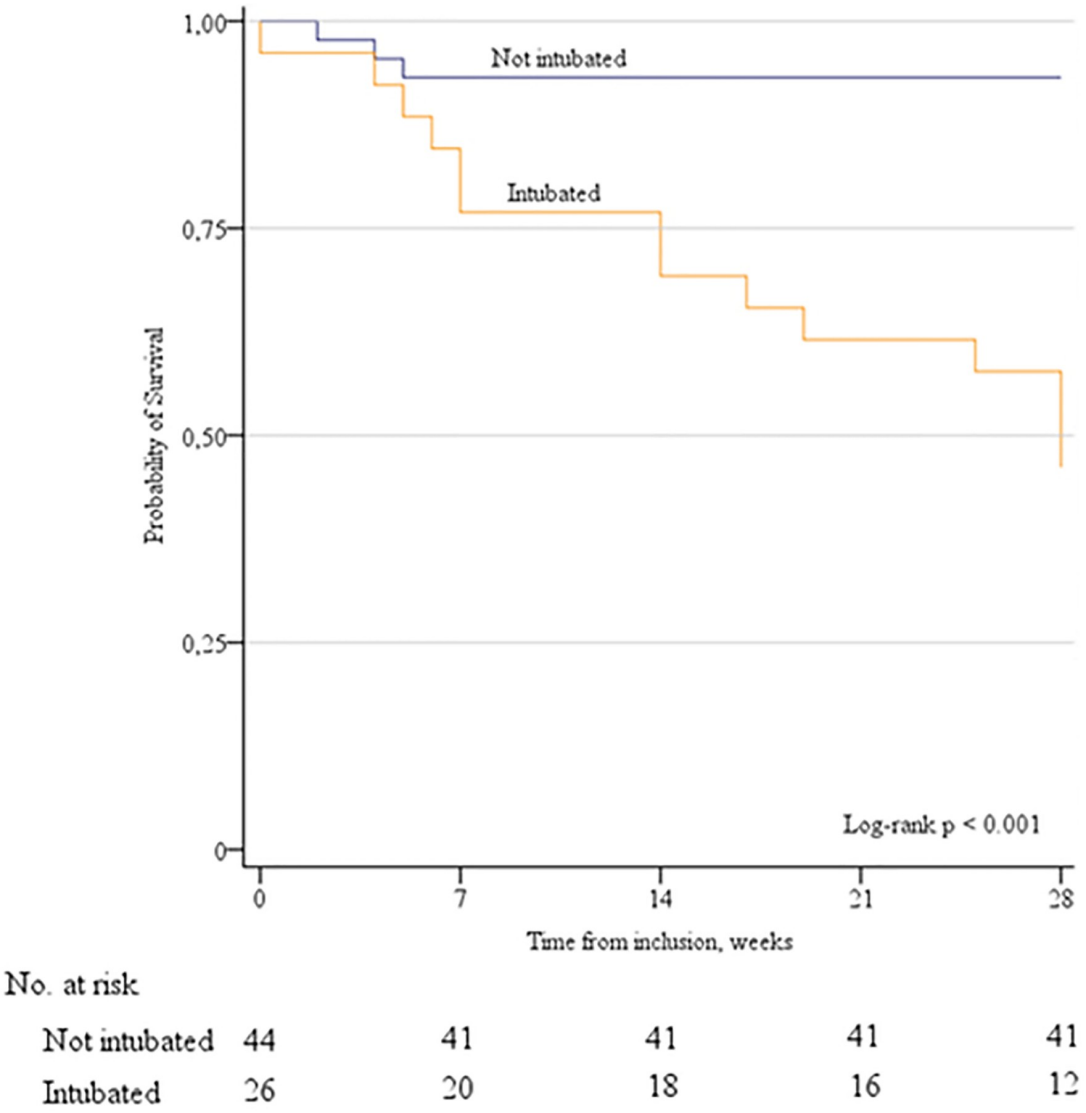
Probability of survival at 28 days.

Deceased patients were older (69 vs 57 years) and had several comorbidities (Charlson Index 6 vs 2 points) (p <0.05), lower pronation rate (17.6% vs 54.7%), greater use of betaferon (52.9 vs 24.5), lower use of cyclosporine (17.6% vs. 50.9%) and hydroxychloroquine (88.2% vs. 100%) (p <0.05). The incidence of major complications in deceased patients was higher (87.5% vs. 45.5%) (p <0.05). No differences were observed in the type of support, the ventilation parameters, or other clinical and biochemical variables. The unadjusted HR for death at 28 days in the group of patients who required ETI was 9.6 (95% CI 2.7 to 33.7; p <0.001). This higher risk of death remained significant after adjusting for age, Charlson index, Pa_O2_/Fi_O2_, SAPS II, pronation, betaferon use, cyclosporine, hydroxychloroquine, and major medical complications (HR 5.4, 95% CI 1.51 to 19.5; p = 0.009) (Fig 3) (S2 Table).

## Discussion

The main finding of this study is that in 37.1% of patients with severe respiratory failure due to COVID-19 managed with non-invasive respiratory support in the IRCU ETI was needed, observing a significantly lower complication and death rates at 28 days in patients were intubation could be avoided. No therapeutic measure except PP was independently associated with intubation rate.

The efficacy of NIV in patients with de novo acute hypoxemic respiratory failure is controversial, which is why the ERS/ATS guideline only recommends its use in selected cases and by expert indication [14]. In this line, in terms of endotracheal intubation, a higher failure rate of NIV compared to oxygen therapy alone has been reported [6], attributed in part to the difficulty of avoiding ventilation-induced lung damage, [15] only achieved in 23% of cases as described by Carteaux et al. [16].

The need for ETI is understood as a severity marker that could contribute to an increased risk of complications and mortality. ETI rates in this study are similar to those previously published [17, 18] although rates may vary widely from 15% [4] to 88% [19]. However, avoiding intubation in patients with severe respiratory failure is of utmost importance given the significant differences in terms of mortality [5, 6, 20] and associated medical complications [21]. In this study, we observed that intubation was independently associated with 28-day mortality after multivariate adjustment, as well as with a higher proportion and frequency of medical complications. The mortality rate of the patients requiring intubation was 53.8% compared to 6.8% in those in whom it could be avoided. In a meta-analysis that included 45 articles with data from 4203 patients with COVID-19, the pooled rate of mortality was 4.3% (95% CI 1.0 to 9.1) [22]. However, for patients with COVID-19 who require ICU admission, mortality rates between 16% [23] and 78% [24] have been described.

The results of this study show that PP is associated with a reduction in the need for intubation, a consistent finding in the literature [20, 25]. In a cohort study that included 20 patients with moderate or severe viral pulmonary ARDS that required support with NIV and HFNC, pronation was found to significantly improve oxygenation, as well as avoid intubation in 55% of cases [20].

The influence of the immune response to SARS-CoV-2 infection plays a key role in the severity of the disease [26]. In this line, in patients in which the local immune response is unable to resolve the infection, a dysfunctional reaction occurs that triggering a cytokine storm [27]. In these patients, high levels of plasma inflammatory mediators, including IL-6, can be observed [28, 29]. In this study, we observed that elevated plasma IL-6 levels on admission were independently associated with an increased risk of intubation. However, we did not observe a significant difference in the rate of intubation or mortality in patients with elevated IL-6 levels who received tocilizumab, an IL-6 antagonist whose efficacy is being currently evaluated in a clinical trial [30]. Although a benefit of corticosteroids in patients with COVID-19 in terms of oxygenation, symptoms, and improvement on chest CT-scan has been reported [31], we did not observe a significant difference in the rate of intubation or mortality in patients not treated with corticosteroids from those who received intravenous methylprednisolone at a dose of 1–2 mg·kg^-1^·day^-1^ or 250 mg boluses.

We recognize that this study has several limitations. First, the findings concerning IL-6, procalcitonin and PaO2/FiO2 ratio must be interpreted with caution because of lost data, which was assumed to be missing at random. Second, selection bias was minimized by adhering to the predefined protocol and by comparing the groups using multivariate analysis. Third, no software was used to record the tidal volume of patients treated with NIV, therefore, it could not be guaranteed in which patients a protective volume was maintained. Fourth, the duration and number of prone sessions were not recorded.

## Conclusions

The endotracheal intubation rate in patients with severe respiratory failure from COVID-19 in this study was 37.1%. The management of these patients with non-invasive respiratory support in an Intermediate Respiratory Care Units could reduce the need for intubation and consequently reduce complications and mortality. We suggest early prone positioning as part of the current respiratory therapeutic arsenal to reduce the need for endotracheal intubation.

## Supporting information

S1 Table Treatment protocol for patients with SARS-CoV-2 infection requiring admission to the Intermediate Respiratory Care Unit. (DOCX)

S2 Table Baseline characteristics, type of respiratory support, pharmacological treatment and complications of patients who were deceased or recovered. (DOCX)

S3 Table Clinical and biochemical characteristics of the patients who received prone positioning and those who did not. (DOCX)

S1 Checklist STROBE statement—Checklist of items that should be included in reports of cohort studies. (DOCX)

## References

[1] World Health Organization. WHO Director- General’s opening remarks at the media briefing on COVID-19–11 March 2020. https://www.who.int/dg/speeches/detail/who-director-general-s-opening-remarks-at-the-media-briefing-on-covid-19—11-march-2020.

[2] Enfermedad por el coronavirus (COVID-19). Actualización n° 98. Centro de Coordinación de Alertas y Emergencias Sanitarias. Secretaría general de sanidad. Ministerio de Sanidad. Actualización 7 de mayo de 2020. https://www.mscbs.gob.es/profesionales/saludPublica/ccayes/alertasActual/nCov-China/documentos/Actualizacion_98_COVID-19.pdf

[3] Metlay JP, Waterer GW, Long AC, Anzueto A, Brozek J, Crothers K, et al Diagnosis and Treatment of Adults with Community-acquired Pneumonia. An Official Clinical Practice Guideline of the American Thoracic Society and Infectious Diseases Society of America. Am J Respir Crit Care Med. 2019 10 1;200(7):e45–e67. 10.1164/rccm.201908-1581ST 31573350 PMC6812437

[4] Guan WJ, Ni ZY, Hu Y, Liang WH, Ou CQ, He JX, et al Clinical Characteristics of Coronavirus Disease 2019 in China. N Engl J Med. 2020 4 30;382(18):1708–1720. 10.1056/NEJMoa2002032 32109013 PMC7092819

[5] Patel BK, Wolfe KS, Pohlman AS, Hall JB, Kress JP. Effect of Noninvasive Ventilation Delivered by Helmet vs Face Mask on the Rate of Endotracheal Intubation in Patients With Acute Respiratory Distress Syndrome: A Randomized Clinical Trial. JAMA. 2016 6 14;315(22):2435–41. 10.1001/jama.2016.6338 27179847 PMC4967560

[6] Frat JP, Thille AW, Mercat A, Girault C, Ragot S, Perbet S, et al High-flow oxygen through nasal cannula in acute hypoxemic respiratory failure. N Engl J Med. 2015 6 4;372(23):2185–96. 10.1056/NEJMoa1503326 25981908

[7] Winck JC, Ambrosino N. COVID-19 pandemic and non invasive respiratory management: Every Goliath needs a David. An evidence based evaluation of problems. Pulmonology. 2020 4 27:S2531-0437(20)30093-3. 10.1016/j.pulmoe.2020.04.013 PMC7183996 32362507

[8] Brochard L, Lefebvre JC, Cordioli RL, Akoumianaki E, Richard JC. Noninvasive ventilation for patients with hypoxemic acute respiratory failure. Semin Respir Crit Care Med. 2014 8;35(4):492–500. 10.1055/s-0034-1383863 25111645

[9] Coudroy R, Hoppe MA, Robert R, Frat JP, Thille AW. Influence of Noninvasive Ventilation Protocol on Intubation Rates in Subjects With De Novo Respiratory Failure. Respir Care. 2020 4;65(4):525–534. 10.4187/respcare.07104 31772067

[10] Satou T, Imamura H, Mochiduki K, Ichikawa M, Takeshige K, Kamijo H, et al Efficacy of protocol-based non-invasive positive pressure ventilation for acute respiratory distress syndrome: a retrospective observational study. Acute Med Surg. 2019 11 6;7(1):e465 10.1002/ams2.465 31988777 PMC6971459

[11] Brower RG, Matthay MA, Morris A, Schoenfeld D, Thompson BT, Wheeler A. Ventilation with lower tidal volumes as compared with traditional tidal volumes for acute lung injury and the acute respiratory distress syndrome. N Engl J Med. 2000 5 4;342(18):1301–8. 10.1056/NEJM200005043421801 10793162

[12] Hui DS, Chow BK, Lo T, Ng SS, Ko FW, Gin T, et al Exhaled air dispersion during noninvasive ventilation via helmets and a total facemask. Chest. 2015 5;147(5):1336–1343. 10.1378/chest.14-1934 25392954 PMC7094250

[13] Cinesi Gómez C, Peñuelas Rodríguez Ó, Luján Torné ML, Egea Santaolalla C, Masa Jiménez JF, García Fernández J, et al Clinical Consensus Recommendations Regarding Non-Invasive Respiratory Support in the Adult Patient with Acute Respiratory Failure Secondary to SARS-CoV-2 infection. Rev Esp Anestesiol Reanim. 2020 5;67(5):261–270. 10.1016/j.redar.2020.03.006 32307151 PMC7161530

[14] Rochwerg B, Brochard L, Elliott MW, Hess D, Hill NS, Nava S, et al Official ERS/ATS clinical practice guidelines: noninvasive ventilation for acute respiratory failure. Eur Respir J. 2017 8 31;50(2):1602426 10.1183/13993003.02426-2016 28860265

[15] Slutsky AS, Ranieri VM. Ventilator-induced lung injury. N Engl J Med. 2013 11 28;369(22):2126–36. 10.1056/NEJMra1208707 Erratum in: N Engl J Med. 2014 Apr 24;370(17):1668–9. 24283226

[16] Carteaux G, Millán-Guilarte T, De Prost N, Razazi K, Abid S, Thille AW, et al Failure of Noninvasive Ventilation for De Novo Acute Hypoxemic Respiratory Failure: Role of Tidal Volume. Crit Care Med. 2016 2;44(2):282–90. 10.1097/CCM.0000000000001379 26584191

[17] Huang C, Wang Y, Li X, Ren L, Zhao J, Hu Y, et al Clinical features of patients infected with 2019 novel coronavirus in Wuhan, China. Lancet. 2020 2 15;395(10223):497–506. 10.1016/S0140-6736(20)30183-5 31986264 PMC7159299

[18] Yang X, Yu Y, Xu J, Shu H, Xia J, Liu H, et al Clinical course and outcomes of critically ill patients with SARS-CoV-2 pneumonia in Wuhan, China: a single-centered, retrospective, observational study. Lancet Respir Med. 2020 5;8(5):475–481. 10.1016/S2213-2600(20)30079-5 32105632 PMC7102538

[19] Grasselli G, Zangrillo A, Zanella A, Antonelli M, Cabrini L, Castelli A, et al Baseline Characteristics and Outcomes of 1591 Patients Infected With SARS-CoV-2 Admitted to ICUs of the Lombardy Region, Italy. JAMA. 2020 4 6;323(16):1574–81. 10.1001/jama.2020.5394 32250385 PMC7136855

[20] Ding L, Wang L, Ma W, He H. Efficacy and safety of early prone positioning combined with HFNC or NIV in moderate to severe ARDS: a multi-center prospective cohort study. Crit Care. 2020 1 30;24(1):28 10.1186/s13054-020-2738-5 32000806 PMC6993481

[21] Kalil AC, Metersky ML, Klompas M, Muscedere J, Sweeney DA, Palmer LB, et al Management of Adults With Hospital-acquired and Ventilator-associated Pneumonia: 2016 Clinical Practice Guidelines by the Infectious Diseases Society of America and the American Thoracic Society. Clin Infect Dis. 2016 9 1;63(5):e61–e111. 10.1093/cid/ciw353 27418577 PMC4981759

[22] Zhang JJY, Lee KS, Ang LW, Leo YS, Young BE. Risk Factors of Severe Disease and Efficacy of Treatment in Patients Infected with COVID-19: A Systematic Review, Meta-Analysis and Meta-Regression Analysis. Clin Infect Dis. 2020 5 14:ciaa576. 10.1093/cid/ciaa576 32407459 PMC7239203

[23] Wang D, Hu B, Hu C, Zhu F, Liu X, Zhang J, et al Clinical Characteristics of 138 Hospitalized Patients With 2019 Novel Coronavirus-Infected Pneumonia in Wuhan, China. JAMA. 2020 2 7;323(11):1061–9. 10.1001/jama.2020.1585 32031570 PMC7042881

[24] Zhou F, Yu T, Du R, Fan G, Liu Y, Liu Z, et al Clinical course and risk factors for mortality of adult inpatients with COVID-19 in Wuhan, China: a retrospective cohort study. Lancet. 2020 3 28;395(10229):1054–1062. 10.1016/S0140-6736(20)30566-3 32171076 PMC7270627

[25] Taccone P, Pesenti A, Latini R, Polli F, Vagginelli F, Mietto C, et al Prone positioning in patients with moderate and severe acute respiratory distress syndrome: a randomized controlled trial. JAMA. 2009 11 11;302(18):1977–84. 10.1001/jama.2009.1614 19903918

[26] McGonagle D, Sharif K, O’Regan A, Bridgewood C. The Role of Cytokines including Interleukin-6 in COVID-19 induced Pneumonia and Macrophage Activation Syndrome-Like Disease. Autoimmun Rev. 2020 6;19(6):102537. 32251717 10.1016/j.autrev.2020.102537 PMC7195002

[27] Ye Q, Wang B, Mao J. The pathogenesis and treatment of the `Cytokine Storm’ in COVID-19. J Infect. 2020 6;80(6):607–613. 10.1016/j.jinf.2020.03.037 32283152 PMC7194613

[28] Tay MZ, Poh CM, Rénia L, MacAry PA, Ng LFP. The trinity of COVID-19: immunity, inflammation and intervention. Nat Rev Immunol. 2020 4 28:1–12. 32346093 10.1038/s41577-020-0311-8 PMC7187672

[29] Wan S, Yi Q, Fan S, Lv J, Zhang X, Guo L, et al Relationships among lymphocyte subsets, cytokines, and the pulmonary inflammation index in coronavirus (COVID-19) infected patients. Br J Haematol. 2020 5;189(3):428–437. 10.1111/bjh.16659 32297671 PMC7262036

[30] Chinese Clinical Trial Register. Chictr.org.cn http://www.chictr.org.cn/showprojen.aspx?proj=49409

[31] Wang Y, Jiang W, He Q, Wang C, Wang B, Zhou P, et al. Early, low-dose and short-term application of corticosteroid treatment in patients with severe COVID-19 pneumonia: single-center experience from Wuhan, China, medRxiv 2020.03.06.20032342.

