# Supplementary material for: Outcomes of an intermediate respiratory care unit in the COVID-19 pandemic
Source: PLoS One. 2020 Dec 16;15(12):e0243968. doi: 10.1371/journal.pone.0243968 (PMC7743985; doi:10.1371/journal.pone.0243968)
Supplement: S1 Table — (DOCX) [file pone.0243968.s001.docx]

**S1 Table. Treatment protocol for patients with SARS-CoV-2 infection requiring admission to the Intermediate Respiratory Care Unit.**

| In all patients admitted to the Intermediate Respiratory Care Unit:  • Lopinavir / ritonavir (200/50 mg tablets), two tablets every 12 hours for seven days. In case of adverse effects, substitution with Darunavir / ritonavir (800/100 mg tablets), one tablet every 24 hours).  • Interferon beta 1β (250 mcg vial), one injection subcutaneously every 48 hours.  • Hydroxychloroquine (200 mg tablets), two tablets every 12 hours on the first day, followed by one tablet every 12 hours for 5 days.  • Ciclosporin A (50 mg and 100 mg capsules), adjusting the dose for weight and kidney function for 14 days.  • Azithromycin (500 mg tablets), one tablet every 24 hours for three to five days.  • Acetylcysteine ​​(300 mg ampoules), two ampoules intravenously every 12 hours in case of accumulation of secretions.  • Enoxaparin (40 mg / 0.4 ml pre-filled syringe) one subcutaneous injection every 24 hours. |
| --- |
| In patients who meet one of the following criteria: (1) increase in Fi_O2_> 0.5, (2) MulBSTA scale ≥ 12 points or (3) CURB65 ≥ 3 points, add:  • Methylprednisolone (250 mg vial) intravenously followed by 3-5 days of methylprednisolone 0.5 mg·kg^-1^·12 hours^-1^.  • Tocilizumab^*^ 400 mg intravenously (if I weigh less than 75 kilograms) or 600 mg (if I weigh more than 75 kilograms). A second dose could be assessed at 12 hours. |

^*^In addition, the patient must meet at least 2 of the following criteria: (1) respiratory rate greater than 30 breaths / minute, (2) need to increase FiO2, (3) D-dimer value greater than 1000 µg / ml or in progressive increase, (3), ferritin value greater than 1000 ng / ml or in progression or (4) interleukin 6 value greater than 40 pg / ml.
