# Supplementary material for: Outcomes of an intermediate respiratory care unit in the COVID-19 pandemic
Source: PLoS One. 2020 Dec 16;15(12):e0243968. doi: 10.1371/journal.pone.0243968 (PMC7743985; doi:10.1371/journal.pone.0243968)
Supplement: S2 Table — (DOCX) [file pone.0243968.s002.docx]

**S2 Table. Baseline characteristics, type of respiratory support, pharmacological treatment and complications of patients who were deceased or recovered.**

|  | Deceased patients (n=17) | Recovered patients (n=53) | p |
| --- | --- | --- | --- |
| Age, years^*^ | 69.0 (59.0 to 76.0) | 57.0 (49.0 to 70.5) | 0.02 |
| Male sex, No. (%) | 13 (76.5) | 41 (77.4) | 1 |
| Body mass index, kg·cm^-2,*^ | 30.4 (29.8 a 33.0) | 31.4 (29.4 to 34.7) | 0.20 |
| Charlson Index score^*^ | 6.0 (3.0 to 7.0) | 2.0 (1.0 to 4.0) | 0.001 |
| Oxygen therapy, No. (%)  High Flow nassal cannula, No. (%)  CPAP, No. (%)  BPAP, No. (%) | 2 (11.8)  11 (64.7)  1 (5.9)  3 (17.6) | 10 (18.9)  28 (52.8)  6 (11.3)  9 (17.0) | 0.71  0.39  1  1 |
| Helmet, No. (%)^§^ | 2 (50.0) | 10 (66.6) | 0.71 |
| CPAP level. cmH_2_O^*^ | 13.0 (13.0 to 13.0) | 15.0 (13.7 to 15.0) | 1 |
| IPAP level, cmH_2_O | 18.3 (2.8) | 18.1 (2.7) | 0.90 |
| EPAP level, cmH_2_O^*^ | 12.0 (10.0 to 12.0) | 10.5 (10.0 to 12.0) | 0.51 |
| Prone positioning, No. (%) | 3 (17.6) | 29 (54.7) | 0.008 |
| Pa_O2_/Fi_O2_, mmHg^*^ | 76.5 (53.2 to 121.5) | 84.0 (55.0 to 143.0) | 0.76 |
| pH | 7.43 (0.07) | 7.42 (0.05) | 0.6 |
| Pa_CO2_, mmHg^*^ | 36.5 (31.5 to 40.0) | 38.0 (34.0 to 42.0) | 0.49 |
| SAPS II score | 37.3 (10.0) | 33.3 (6.9) | 0.14 |
| Lymphocytes cells·L^-1^ | 747 (462) | 937 (433) | 0.12 |
| D dimer, µg·ml^-1,*^ | 316 (294 to 599) | 451 (254 to 943) | 1 |
| Ferritin, ng·ml^-1,*^ | 1339 (673 to 6259) | 1371 (712 to 2158) | 0.95 |
| Procalcitonin, ng·ml^-1,*^ | 0.23 (0.17 to 0.56) | 0.17 (0.06 to 0.27) | 0.09 |
| Interleukin 6, pg·ml^-1,*^ | 277 (42 to 2331) | 58 (29 to 110) | 0.72 |
| Treatment received  Acetylcysteine, No (%)  Azithromycine, No. (%)  Betaferón, No. (%)  Cyclosporine, No. (%)  Hydroxychloroquine, No. (%)  Lopinavir/ritonavir, No. (%)  Methylprednisolone  (bolus 250 mg), No. (%)  Methylprednisolone  (≥ 1 mg·kg^-1^·day^-1^), No. (%)  Tocilizumab, No. (%) | 13 (76.5)  8 (47.1)  9 (52.9)  3 (17.6)  15 (88.2)  17 (100.0)  6 (35.3)  13 (76.5)  10 (58.8) | 31 (58.5)  14 (26.4)  13 (24.5)  27 (50.9)  53 (100.0)  51 (96.2)  27 (50.9)  38 (71.7)  39 (73.6) | 0.18  0.11  0.02  0.01  0.01    1  0.26  1  0.24 |
| Major complications, No. (%) | 12 (70.6) | 29 (54.7) | 0.16 |
| Number of major complications^£^ | 3.5 (0.2 to 7.0) | 1.0 (0.0 to 2.0) | 0.21 |

CPAP: continuous positive airway pressure, BPAP: bilevel positive airway pressure, IPAP: inspiratory positive airway pressure, EPAP: Expiratory Positive Airway Pressure, SAPS II: Simple Acute Physiologic Score II.

^*^Data expressed as median (interquartile range).

^§^From the total of patients who received non-invasive ventilation (CPAP or BPAP mode).
