# Supplementary material for: Outcomes of an intermediate respiratory care unit in the COVID-19 pandemic
Source: PLoS One. 2020 Dec 16;15(12):e0243968. doi: 10.1371/journal.pone.0243968 (PMC7743985; doi:10.1371/journal.pone.0243968)
Supplement: S3 Table — (DOCX) [file pone.0243968.s003.docx]

**S3 Table. Clinical and biochemical characteristics of the patients who received prone positioning and those who did not.**

|  | Prone positioning  received  (n=32) | Prone positioning  not received  (n=38) | p-value |
| --- | --- | --- | --- |
| Age, years* | 60.0 (50.7 to 71.2) | 64.5 (51.7 to 74.0) | 0.052 |
| Male sex, No. (%) | 27 (84.4) | 27 (71.1) | 0.18 |
| Body mass index, kg·m^-2.^* | 31.3 (28.4 to 33.8) | 31.2 (29.8 to 35.1) | 1 |
| Charlson Index score* | 2.0 (1.0 to 3.0) | 3.0 (1.0 to 5.0) | 0.16 |
| Pa_O2_/Fi_O2_, mmHg* | 74.0 (55.0 to 118.0) | 106.0 (55.5 to 143.7) | 0.32 |
| pH | 7.42 (0.04) | 7.43 (0.07) | 0.71 |
| Pa_CO2_, mmHg* | 38.0 (34.0 to 44.0) | 37.0 (33.5 to 40.0) | 0.65 |
| SAPS II score | 32.8 (6.6) | 35.6 (8.8) | 0.14 |
| Lymphocytes cells·L^-1^ | 903 (461) | 881 (436.7) | 0.84 |
| D dimer, μg·ml^-1,^* | 416 (264 to 825) | 559 (252 to 946) | 0.91 |
| Ferritin, ng·ml^-1,^* | 1444 (796 to 2877) | 782 (610 to 2002) | 0.57 |
| Procalcitonin, ng·ml^-1,^* | 0.16 (0.06 to 0.29) | 0.19 (0.11 to 0.34) | 0.44 |
| Interleukin 6, pg·ml^-1,^* | 57.2 (32.0 to 151.2) | 75.1 (17.0 to 155.5) | 1 |

SAPS II, Simple Acute Physiologic Score II.

*Data expressed as median (interquartile range).
