# Supplementary material for: Outcomes of an intermediate respiratory care unit in the COVID-19 pandemic
Source: PLoS One. 2020 Dec 16;15(12):e0243968. doi: 10.1371/journal.pone.0243968 (PMC7743985; doi:10.1371/journal.pone.0243968)
Supplement: S1 Checklist — (DOCX) [file pone.0243968.s004.docx]

STROBE Statement—Checklist of items that should be included in reports of ***cohort studies***

|  | Item No | Recommendation |
| --- | --- | --- |
| **Title and abstract** | 1 | (*a*) Outcomes of an Intermediate Respiratory Care Unit in the COVID-19 pandemic |
|  |  | (*b*) RATIONALE: 15% of COVID-19 patients develop severe pneumonia. Non-invasive mechanical ventilation and high-flow nasal cannula can reduce the rate of endotracheal intubation in adult respiratory distress syndrome, although failure rate is high.  OBJECTIVE: To describe the rate of endotracheal intubation, the effectiveness of treatment, complications and mortality in patients with severe respiratory failure due to COVID-19.  METHODS: Prospective cohort study in a first-level hospital in Madrid. Patients with a positive polymerase chain reaction for SARS-CoV-2 and admitted to the Intermediate Respiratory Care Unit with tachypnea, use of accessory musculature or SpO2 <92% despite FiO2> 0.5 were included. Intubation rate, medical complications, and 28-day mortality were recorded. Statistical analysis through association studies, logistic and Cox regression models and survival analysis was performed.  RESULTS: Seventy patients were included. 37.1% required endotracheal intubation, 58.6% suffered medical complications and 24.3% died. Prone positioning was independently associated with lower need for endotracheal intubation (OR 0.05; 95% CI 0.005-0.54, p=0.001). The adjusted HR for death at 28 days in the group of patients requiring endotracheal intubation was 5.4 (95% CI 1.51-19.5; p=0.009).  CONCLUSIONS: The rate of endotracheal intubation in patients with severe respiratory failure from COVID-19 was 37.1%. Complications and mortality were lower in patients in whom endotracheal intubation could be avoided. Prone positioning could reduce the need for endotracheal intubation. |
| Introduction | | |
| Background/rationale | 2 | On March 11, 2020, the World Health Organization declared the novel COVID-19 outbreak a global pandemic (1). More than three million cases had been reported worldwide by the end of the first week in May, of which 221,000 had been declared in Spain (2). The spectrum of this disease caused by the SARS-CoV-2 coronavirus ranges from a common cold to a severe pneumonia defined according to American Thoracic Society criteria (3) in a not negligible 15% of patients (4). In our setting, the rapid increase in the incidence of COVID-19 and consequent saturation of the capacity of the intensive care units (ICUs) led to a significant role for intermediate respiratory care units (IRCUs) in the management of these patients, with the principal purpose of reducing the need for endotracheal intubation (ETI) using non-invasive respiratory support.  The efficacy of high-flow nasal cannula (HFNC) and non-invasive mechanical ventilation (NIV) in adult respiratory distress syndrome (ARDS) have been previously investigated as therapies that could reduce intubation rate and mortality (5-7). However, the use of these respiratory support therapies beyond the stablished time or severity window of the ARDS could lead to an increase in mortality (8), with failure rates in moderate or severe forms between 38% and 80% respectively (9,10). Hence the importance of adequate selection of patients and early access to ETI in the absence of response. |
| Objectives | 3 | The objective of the study is to describe the ETI rate in patients with severe respiratory failure due to COVID-19 managed in an IRCU, the efficacy of the respiratory support and pharmacological treatments, and the medical complication and mortality rates |
| Methods | | |
| Study design | 4 | Prospective cohort study |
| Setting | 5 | Study conducted in an 11-bed IRCU led by a team of pneumologists with support from intensive care and anesthesia specialists in the first-level Infanta Elena University Hospital, Madrid (Spain). |
| Participants | 6 | (*a*) Inclusion criteria were the following: adult patients with a positive PCR for COVID-19 and admission to the IRCU with at least one of the following: respiratory rate (RR) > 30 breaths·minute-1, severe dyspnea, use of accessory muscles or SpO2 <92% despite FiO2 >0.5 oxygen therapy. Patients were included between March 6 and April 8, 2020 and were follow-up for a 28-day period. Patients not candidate for ETI according to the ethics committee document created and approved in March 2020 and patients transferred from the ICU to undergo weaning were excluded from the study |
|  |  | (*b*) Does not apply |
| Variables | 7 | Upon admission to the IRCU, a chest radiograph was performed and a blood sample was taken for the analysis of pH, partial pressure of O2 (PaO2) and CO2 (PaCO2), and a complete biochemistry including creatinine, urea, bilirubin, liver enzymes, profile ionic, ferritin, procalcitonin, interleukin 6 (IL-6), blood count and coagulation. Age, sex, body mass index (BMI), the Charlson index and the Simplified Acute Physiology Score II (SAPS II), the choice of treatment, the highest degree of respiratory support required, the parameters of PEEP, SP and whether the patient was set in the prone position (PP) were recorded. The incidence and number of grade III, IV, and V medical complications were recorded according to the Common Terminology Criteria for Adverse Events (CTCAE) v5.0, as well as the intubation rate, and 28-day mortality.  Specific COVID-19 pharmacological treatment choice was stablished at the discretion of the prescribing specialist following the center's protocol.  In patients presenting a SpO2 <92% despite FiO2> 0.5 without a RR of > 30 breaths·minute-1 or use of accessory muscles upon admission to the IRCU, treatment with HFNC (AIRVO 2, Fisher and Paykel healthcare) was started with an initial flow of 60 liters/minute, a temperature of 37.0 ºC and a FiO2 between 0.5 and 1 with the objective of a SpO2> 92%.  In patients with RR> 30 breaths·minute-1, severe dyspnea or use of accessory muscles, support was started with IRCU or home ventilators (V60 Philips Respironics, Vivo 55 Breas, Vivo 60 Breas and Astral 150 Resmed) in CPAP or bilevel pressure mode, titrating the positive end expiratory pressure (PEEP) to achieve an SpO2> 92% with the lowest possible FiO2 and the support pressure (SP) to reduce the RR <30 breaths·minute-1, the use of accessory muscles and the degree of dyspnea.  In all cases, it was considered a priority to achieve a tidal volume of less than 6-8 ml·min-1·kg-1 of ideal body weight (11). The use of Helmet was preferred for its advantages in terms of efficacy (5) and safety (12) in accordance with the recommendations of our scientific society (13).  At the discretion of the prescribing clinician, the patient was pronated in the first 12 hours of admission to the IRCU between 1 and 3 times per day for 60 minutes or as long as the patient could tolerate.  Discharge to conventional hospitalization occurred when the patient presented an SpO2> 92% with FiO2 <0.5, RR <30/min, and had no evidence of dyspnea or use of accessory muscles. ETI was performed if SpO2 <88%, RR> 35 breaths·minute-1, impaired level of consciousness or hemodynamic instability, despite 1-2 hours of respiratory support with a FiO2> 0.5. |
| Data sources/ measurement | 8* | A chest radiograph was performed.  Blood sample was taken for the analysis of pH, partial pressure of O2 (PaO2) and CO2 (PaCO2), and a complete biochemistry including creatinine, urea, bilirubin, liver enzymes, profile ionic, ferritin, procalcitonin, interleukin 6 (IL-6), blood count and coagulation.  Age, sex, body mass index (BMI), the Charlson index and the Simplified Acute Physiology Score II (SAPS II), the choice of treatment, the highest degree of respiratory support required, the parameters of PEEP, SP and whether the patient was set in the prone position (PP) were recorded |
| Bias | 9 | Statistical analysis through logistic and Cox regression models |
| Study size | 10 | Assuming an intubation rate of 60% (9,10) in patients with moderate or severe ARDS treated with HFNC or NIV, we calculated that a sample size of at least 59 patients would be required with a two tail alpha risk of 0.05 and a statistical power of 80% in order to detect a difference in the ETI rate of 20%, which we consider clinically relevant. A follow-up loss rate of 20% was estimated |
| Quantitative variables | 11 | The Shapiro-Wilk test was used to verify the normality. Normally distributed quantitative variables are expressed as mean (standard deviation) or as median (interquartile range).  Categorical variables are expressed as absolute and percentage value. |
| Statistical methods | 12 | (*a*) The characteristics of the groups of intubated and non-intubated patients and deceased and non-deceased patients were compared using the Student's t-test and the Mann-Whitney U test for quantitative variables and the Chi-square test and Fisher's exact test for categorical variables. Multivariate logistic regression was performed to determine whether there were any independent predictors of ETI. Kaplan-Meier survival curves were constructed and a log-rank analysis was conducted. We determined predictors of 28-day mortality rate using Cox proportional hazard modelsfor confounding |
|  |  | (*b*) Does not apply |
|  |  | (*c*) Missing data were excluded from the statistical analysis, being specified as missing value in the SPSS program. |
|  |  | (*d*) Does not apply |
|  |  | (*e*) Does not apply |
| Results | | |
| Participants | 13* | (a) From March 6 to April 8, 2020, 93 patients were screened, 22 patients were excluded because they were not candidates for endotracheal intubation and 1 because they were transferred from the ICU. 70 patients were follow-up for a 28-day period and were finally included in the analysis. |
|  |  | (b) Patients not candidates for endotracheal intubation were excluded because the endotracheal intubation rate was the main objective of the study.  Patients transferred from the ICU were excluded because they were in a recovery phase of the disease not representative of our main objective. |
|  |  | (c) A flow diagram was used (see figure 1). |
| Descriptive data | 14* | (a) Characteristics of study and information on exposures and potential confounders were given. See table 1 and paragraphs 2, 3 and 4 in the results section. |
|  |  | (b) Number of participants (70) with missing data for:  Age: 0.  Sex: 0.  BMI (body mass index): 18.  Charlson index: 0.  Pa/FiO2: 15.  pH: 21.  PaCO2: 21.  SAPS II: 2.  Lymphocytes: 1.  Ferritin: 29.  D dimer: 19.  Interleukin 6: 13.  Procalcitonin: 11.  Oxygen therapy: 0.  High Flow nassal cannula: 0.  CPAP: 0.  BPAP: 0.  Helmet: 0.  CPAP: 0.  IPAP: 0.  Prone positioning: 0.  Acetylcysteine: 0.  Azithromycine: 0.  Betaferón: 0.  Cyclosporine: 0.  Hydroxychloroquine: 0.  Lopinavir/ritonavir: 0.  Methylprednisolone (bolus 250 mg): 0.  Methylprednisolone (≥ 1 mg·kg-1·day^-1^): 0.  Tocilizumab: 0.  Myocardic injury: 0.  Highest cTnT value: 0.  QT prolongation: 0.  De novo supraventricular tachycardia: 0.  Acute pulmonary embolism: 0.  Hypertension: 0.  Hypotension: 0.  Acute kidney failure: 0.  Bacteraemia: 0.  Septic shock: 0.  Hospital acquired pneumonia: 0.  Intravascular catheter infection: 0.  Fungal infection: 0.  Cytomegalovirus infection: 0.  Agitation: 0.  Seizure: 0.  Bronchial obstruction: 0.  Pneumothorax: 0.  Abnormal liver function: 0.  Hyoperkalemia: 0.  Hypokalemia: 0.  Hypernatremia: 0.  Hyponatremia: 0.  Hypocalcemia: 0.  Anemia: 0.  Thrombocytopenia: 0.  Gastrointestinal bleeding: 0.  Skin ulceration: 0.  Major complications: 0.  Number of major complications: 0.  Endotracheal intubation: 0.  Mortality: 0. |
|  |  | (c) Follow up-time: mean 54.7 ± 18.3 (SD), percentile (100): 64. |
| Outcome data | 15* | See paragraph 2, 4, 5 and table 3 in the results section. |
| Main results | 16 | (*a*) A logistic regression model was designed to search for predictive variables of ETI following the backwards method. The unadjusted OR for the association between prone positioning and ETI was 0.20, 95% CI 0.07 to 0.61; p = 0.005). The variables age, sex, PaO2/FiO2, lymphocytes, D-dimer, procalcitonin, IL-6, pronation, the use of acetylcysteine, azithromycin, betaferon and cyclosporin were included in the model. PP was observed to be independently associated with the need for intubation (adjusted OR of 0.05, 95% CI 0.005 to 0.54, p = 0.001) (the baseline characteristics of patients who received PP and those who did not are summarized in the supplementary table S3) as well as IL-6 values greater than 1000 pg/ml (adjusted OR of 65.2, IC95 % 3.5 to 1198, p = 0.005). The unadjusted OR for the association between IL-6 values greater than 1000 and ETI was 7.1, 95% CI 1.9 to 26.1; p = 0.005).  The unadjusted HR for death at 28 days in the group of patients who required ETI was 9.6 (95% CI 2.7-33.7; p <0.001). This higher risk of death remained significant after adjusting for age, Charlson index, PaO2/FiO2, SAPS II, pronation, betaferon use, cyclosporine, hydroxychloroquine, and major medical complications (HR 5.4, 95% CI 1.51-19.5; p = 0.009). |
|  |  | (*b*) Age (> 60 years), Pa/FiO2 (<100), lymphocytes (<900), D-Dimer (>1500), procalcitonin (>0.2), IL-6 (>1000). |
|  |  | (*c*) If relevant, consider translating estimates of relative risk into absolute risk for a meaningful time period |
| Other analyses | 17 | Does not apply |
| Discussion | | |
| Key results | 18 | 37.1% of patients required ETI, 58.6% of patients suffered major medical complications, and mortality was 24.3%.  No significant differences were observed in the type of respiratory support received, the ventilation parameters, the type of interface or in the rest of the pharmacological treatment used.  Patients who required endotracheal intubation suffered a higher incidence and number of complications (p <0.05), with a higher proportion of myocardial injury, hypertension, acute kidney failure, bacteremia, septic shock, nosocomial pneumonia, bronchial obstruction, anemia, thrombopenia, and cutaneous ulcers.  The mortality in patients who required intubation was significantly higher than those who did not require intubation (p <0.001) |
| Limitations | 19 | We recognize that this study has several limitations. First, the findings concerning IL-6, procalcitonin and PaO2/FiO2 ratio must be interpreted with caution because of lost data, which was assumed to be missing at random. Second, selection bias was minimized by adhering to the predefined protocol and by comparing the groups using multivariate analysis. Third, no software was used to record the tidal volume of patients treated with NIV, therefore, it could not be guaranteed in which patients a protective volume was maintained. Fourth, the duration and number of prone sessions were not recorded. |
| Interpretation | 20 | The management of these patients with non-invasive respiratory support in an Intermediate Respiratory Care Units could reduce the need for intubation and consequently reduce complications and mortality. We suggest early prone positioning as part of the current respiratory therapeutic arsenal to reduce the need for endotracheal intubation.. |
| Generalisability | 21 | We consider that the external validity is high since the patients included in the analysis correctly represent the population, the study design is prospective, there were no losses to follow-up, and multivariate regression models were conducted. |
| Other information | | |
| Funding | 22 | The authors were not funded to undertake this study |

*Give information separately for exposed and unexposed groups.
